# Identification of G-quadruplex anti-Interleukin-2 aptamer with high specificity through SELEX stringency

**DOI:** 10.1016/j.heliyon.2022.e09721

**Published:** 2022-06-15

**Authors:** Mohsen Momeni, Kazem Mashayekhi, Jamshid Gholizadeh Navashenaq, Mojtaba Sankian

**Affiliations:** a Immuno-Biochemistry Lab, Immunology Research Center, Mashhad University of Medical Sciences, Mashhad, Iran; b Immunology of Infectious Diseases Research Center, Research Institute of Basic Medical Sciences, Rafsanjan University of Medical Sciences, Rafsanjan, Iran; c Noncommunicable Diseases Research Center, Bam University of Medical Sciences, Bam, Iran

**Keywords:** Interleukin-2, Aptamer, SELEX stringency, G-quadruplex, Dot blot assay

## Abstract

Aptamers are short single-stranded oligonucleotides capable of binding to various targets with high specificity and affinity. This study aimed to identify an aptamer against mouse interleukin-2 (mIL-2) as one of the most important cytokines in autoimmune diseases for diagnostic and therapeutic purposes. For this purpose, 14 SELEX rounds were performed on recombinant mIL-2 with high stringency. The dot blot and flow cytometry techniques were conducted to determine affinity, dissociation constant (K_d_), specificity, and SELEX rounds screening. The stringency of rounds was considered based on aptamer/target incubation time, washing steps, and target proteins. Finally, the aptamer's structure was mapped and predicted by M-fold and QGRS Mapper web-based software. After 14 rounds, the flow cytometry analysis revealed that the 11^th^ round was a proper round. The high-affinity aptamers M20 and M15 were chosen for their ability to bind mIL-2. According to DNA folding software, M20 and M15 aptamers had G-quadruplex and stem-loop structures, respectively. The M20 aptamer affinity was greater than M15, and its predicted K_d_ was 91 nM. A simple SELEX protocol with round stringency was explained to identify DNA aptamers against protein targets. The reported G-quadruplex aptamer might have potential diagnostic or therapeutic application in IL-2–related disorders.

## 1 Introduction

In the past few decades, the identification and detection of various immune system proteins, including cytokines, has been particularly noted. Besides the importance of identifying these proteins in the course of diseases, it is also important to target them to treat diseases such as allergies, autoimmune diseases, infectious diseases, and cancers [1, 2, 3]. Interleukin-2 (IL-2), as an autocrine growth factor of the activated T-cell, is a promising target molecule in modulating immune response [4, 5], and there is significant interest in the inhibition of IL-2 as a treatment method [6]. For this purpose, antibodies (Abs) are well-known agents for inhibiting cytokines. Despite their numerous applications, antibodies have a number of limitations, including in vivo production, instability, high toxicity, and immunogenicity [7, 8].

The necessity of new laboratory methods for identifying ligands with high affinity has led to the creation of aptamers for new therapeutic and diagnostic approaches [9, 10]. Aptamers are 3D (3-dimensional)-structured and in vitro-chemically synthesized of short oligonucleotides of ssDNA or ssRNA, which could bind against many targets such as ions, antibiotics, and amino acids, as well as large protein complexes, enzymes, antibodies, and cell surface receptors with high affinity and specificity [11, 12].

The selection of aptamers *in vitro* from a nucleic acid library of DNA or RNA is performed during a repetitive process named SELEX (Systematic Evolution of Ligands by Exponential Enrichment) [13, 14, 15]. The SELEX process is a powerful combination chemistry process in which aptamers with the maximum binding affinity and specificity are generated against the target [15]. Aptamer-based therapies have many advantages in comparison with protein-based ones such as Abs. For example, lack of immunogenicity and accumulation in organs, small size, and fast blood clearance. This study aimed to identify DNA aptamers with high affinity and specificity against mIL-2 for diagnostic or therapeutic purposes using a convenient and simple SELEX protocol with high stringency.

## 2 Materials and methods

### 2.1 Materials

*mIL-2 cloning:* The recombinant mIL-2 plasmid with a 23 kDa molecular weight was used in our previous study [4]. The plasmid extraction kit and protein purification columns were purchased from Parstous Co. (Mashhad, Iran).

*DNA library and primers*: The properties of the library were as follows: 79 mer length, a region with 42 random nucleotides, and two fixed-primer sites (5’-GCTGTGTGACTCCTGCAA-42 random region -GCAGCTGTATCTTGTCTCC-3’). The 5’-biotinylated and fluorescent sense primer were used to determination of aptamer binding affinity and screening of pools (5’-Biotin or FAM-GCTGTGTGACTCCTGCAA-3’). To make single-stranded aptamers from double-stranded forms with lambda exonuclease enzyme (ThermoScientific, Germany), 5’-phosphorylated antisense primer (5’-phosphate-GGAGACAAGATACAGCTGC-3’) was used. All primers and libraries were obtained from Bioneer Co. (Shanghai, China).

*SELEX process*: PCR reagents, Taq polymerase, Klentaq polymerase, and silica nanobeads for ssDNA purification were purchased from Parstous Co. (Mashhad, Iran). The yeast tRNA and TA-clone PCR kit were purchased from Sigma (Missouri, USA) and Fermentas (Wisconsin, USA), respectively. The Ni-NTA agarose magnetic beads were obtained from Qiagen Co. (Hilden, Germany).

*SELEX rounds screening and binding affinity:* To round screening, the flow cytometry device FACSCalibur (BD Biosciences, CA, USA) was used. The chemiluminescence substrate, polyvinylpyrrolidone (MW 40000 PVP), nitrocellulose membrane, and HRP-streptavidin conjugate were purchased from Parstous Co. (Mashhad, Iran), Sigma-Aldrich Co. (Darmstadt, Germany), Bio-Rad Co. (California, USA), and BD Pharmingen Co. (California, USA), respectively.

### 2.2 The first SELEX round step

The mIL-2 was expressed in our previous study [4]. Purified mIL-2 was dialyzed against phosphate buffer (with 300 mM NaCl, pH 7.4). The purity and concentration of protein were evaluated by SDS-PAGE analysis. Firstly, 10 μl of Ni-NTA beads was mixed with 100 pmol of mIL-2 in 100 μl phosphate buffer (with 500 mM NaCl, pH 7.4) for 1 h with shaking at room temperature. The bead/mIL-2 complex was washed two times with 100 μl phosphate buffer (with 150 mM NaCl, pH 7.4). Next, about 1 nmol of DNA library was added to the 200 μl of aptamer binding buffer (50 mM Tris-HCl, 100 mM NaCl, 5 mM KCl, 1 mM MgCl_2_, pH:7.4), and incubated at 95 °C for 10 min, then placed on quick crash-ice for 10 min. A treated DNA library with BSA 1% was added to the bead/mIL-2 complex and incubated for 1 h with shaking at room temperature. The bead/mIL-2/aptamer complex was washed twice for 30 s with 100 μl of aptamer washing buffer (50 mM Tris-HCl, 100 mM NaCl, 1 mM MgCl_2_, 5 mM KCl, pH 7.4). Finally, elution of specific DNA aptamers were performed with 50 μl of elution buffer (20 mM Tris-HCl, 250 mM Imidazole, pH 7.4).

### 2.3 Enrichment of pools by PCR step

To enrichment of aptamers in eluted pools, a PCR technique was performed. Different PCR cycles were conducted in each round to determine the desired cycles for pool enrichment. The PCR cycles condition was as follow: 8, 10, 12, 15, 18, 20 and 25 with Klentaq polymerase (initial denaturation: 94 °C for 3 min, denaturation: 94 °C for 30 s, annealing/extension: 63 °C for 45 s, and final extension: 72 °C for 3 min).

### 2.4 The ssDNA purification and preparation step

In each round, eluted pools were phosphorylated by a 5’-phosphorylated primer in suitable PCR cycles, then single-strand DNA was prepared for the next rounds. Next, the lambda exonuclease method was performed to digest double-strand DNA pools. Finally, purification of ssDNA was conducted with silica nanobeads. To start the next rounds, total ssDNA aptamer pools were used.

### 2.5 Counter SELEX step and continue rounds

**Figure 1:**
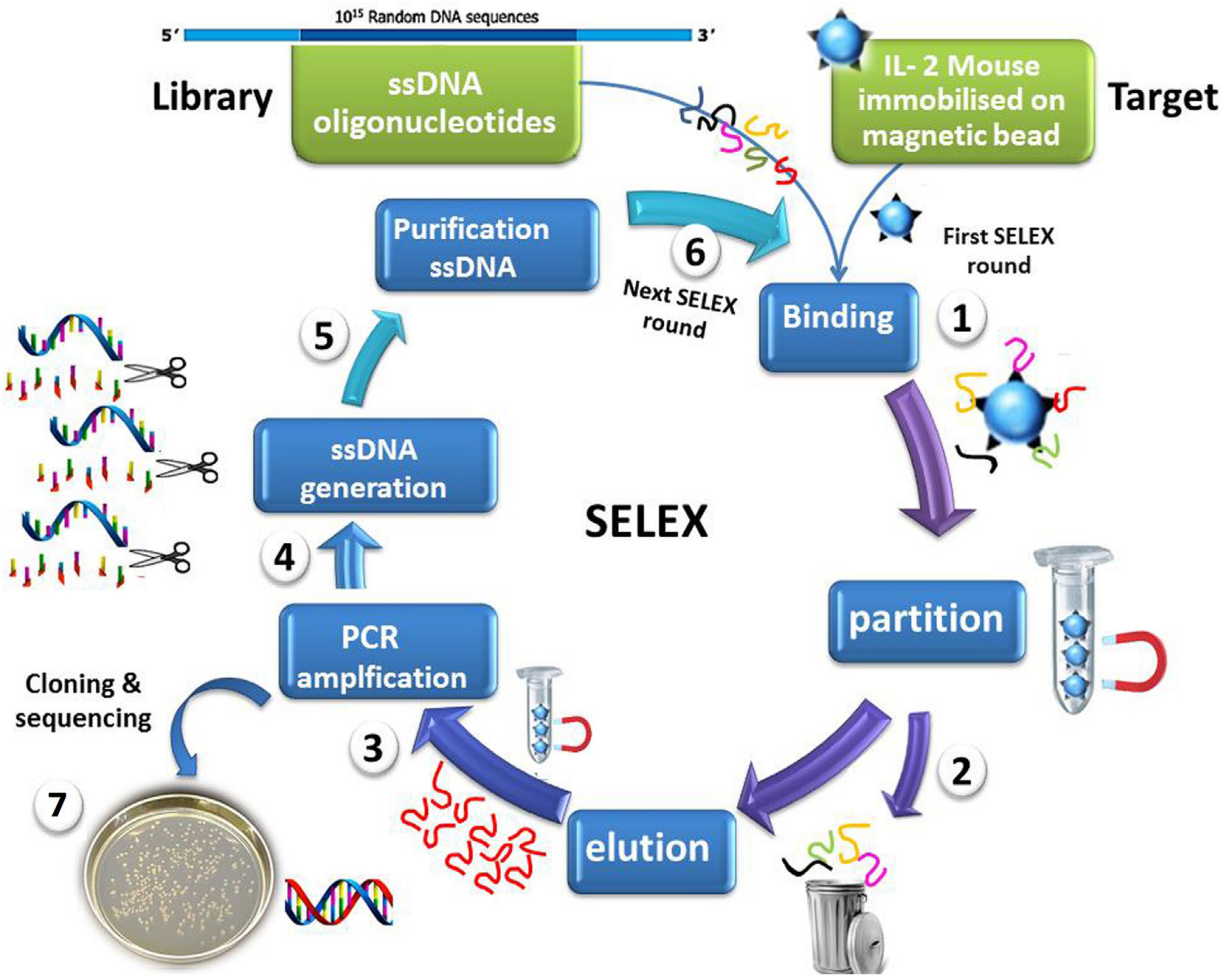
Schematic figure of the SELEX process: The mIL-2 was immobilized on magnetic beads and then shaped ssDNA library added to mIL-2/bead complex (Step 1). The unbound aptamers were removed (Step 2). Next, the mIL-2/aptamer complex was eluted, and specific aptamers were amplified by PCR (Step 3). The amplified specific aptamers were digested by the lambda exonuclease enzyme (Step 4). The purified-ssDNA was added to the mIL-2/aptamer complex again and started the next round (Steps 5 and 6). Finally, the aptamers from the proper round were chosen for cloning and sequencing (Step 7) (ChemDraw, v.17).

The round stringency was performed after the first round to reach the specific aptamers. The round stringency was considered for mIL-2 concentration, incubation time of mIL-2/aptamer, and mIL-2/aptamer/bead complex washing steps. The counter SELEX step was done on His-tagged protein (recombinant mouse TGF-β1)/bead complex and naked magnetics beads to remove non-specific aptamers. This step was carried out from the 3^rd^ to the 9^th^ round. Then, unattached aptamers in the supernatant were incubated with the mIL-2/aptamer/bead complex. Also, yeast tRNA (0.1 mg/ml = 1%) was added to the 8 last rounds as a non-specific competitor in the aptamer binding buffer, and in the last 5 steps, Tween-20 (0.05%) was used for the elimination of weak aptamers in the aptamer washing buffer. Finally, a total of 14 SELEX rounds were conducted. The conditions of SELEX rounds are described in Table 1 and Figure 1.

**Table 1:** The condition of 14 SELEX rounds.

Rounds	SELEX		Counter SELEX			Washing				Yeast tRNA	Desired PCR cycle
	mIL-2 (pmol)	Time∗∗	His-tag protein (pmol)	Naked Beads (μl)	Time∗∗	No.	Buffer (μl)	Time∗∗	Tween-20 0.05%		
1	100	60	-	-	60	2	100	0.5	-	-	12
2	100	60	-	-		3	100	1	-	-	12
3	100	60	100	-		3	100	1	-	-	12
4	100	45	-	-		3	100	2	-	-	13
5	83	45	100	-		3	100	2	-	-	14
6	83	45	-	10		3	100	2	-	-	14
7	83	30	100	-		3	100	3	-	+	14
8	66	30	-	10		3	100	3	-	+	16
9	66	30	100	-		3	100	3	-	+	16
10	50	20	-	-		3	200	5	+	+	16
11∗	50	20	-	-		3	200	5	+	+	17
12	40	20	-	-		3	200	5	+	+	16
13	40	20	-	-		3	200	5	+	+	17
14	40	20	-	-		3	200	5	+	+	18

(∗Appropriate round, ∗∗ Minute).

### 2.6 The screening of rounds by flow cytometry

The flow cytometry technique was performed for round monitoring in the present study. In the library, aptamers in the rounds of 9^th^, 11^th^, 12^th^, and 14^th^ were chosen to identify desired aptamers. To make fluorescent-labeled aptamer pools, 5’-FAM primer was used. Briefly, about 100 nM ssDNA of the mentioned rounds and library were incubated with the mIL-2/bead complex in 100 μl of aptamer binding buffer for 20 min at room temperature with shaking. Then, the mIL-2/bead complex was washed with aptamer washing buffer, and the complexes were mixed in 300 μl of aptamer binding buffer for flow cytometry analysis. Finally, the mean fluorescence intensity (MFI) of all samples was measured using a flow cytometry device with a 20,000 bead count.

### 2.7 Aptamers cloning, sequencing, and structure

Based on the highest MFI, the best round was selected. Briefly, the ssDNA of the selected round was amplified with the unlabeled forward and reverse primers. TA-cloning method was used for PCR products cloning. Next, to confirm the insertion of aptamers, PCR was performed with M13 and T7 primers on extracted recombinant plasmids. Then, nucleotide sequencing was done on the recombinant plasmids with proper insertion (Macrogen Co., Seoul, South Korea). Plasmids were sequenced based on M13 and T7 primers by the Applied Biosystems 3730XL DNA Analyzer instrument. Aptamer sequences were analyzed with BioEdit alignment software (version 7.2.5). Finally, aptamer structures were mapped and predicted by web-based software (M-fold: www.unafold.rna.albany.edu; QGRS Mapper: www.bioinformatics.ramapo.edu).

### 2.8 Assessment of aptamer K_d_ and specific affinity

In order to determine the aptamer K_d_ and specific binding, the dot blot technique was performed as in the previous study [10]. Briefly, about 300 ng of mIL-2 was coated on nitrocellulose and blocked with PBS buffer containing 0.002% Tween-20, 0.05% BSA, and 1% PVP for 2 h. Next, after washing with PBS-Tween, a biotinylated-aptamer (300 nM) was added to mIL-2 dots and incubated for 1 h at 25 °C, and then washed (PBS-Tween containing 3 mM MgCl_2_). The streptavidin-HRP conjugate (1:15000 diluted in PBS) was added to dots, then incubated for 90 min at 25 °C. Finally, nitrocellulose sheets were assessed by chemiluminescent substrate, and chemiluminescent signals were detected by a chemiluminescent instrument (GBoxChemi HR, Cambridge, UK). His-tagged recombinant mouse TGF-β was used as a negative control in dot blot assay to investigate the specificity of aptamer binding to the target. Likewise, the aptamer K_d_ was calculated. For this purpose, the mIL-2 was prepared at a concentration of 300 ng/μl by dilution with PBS buffer and blotted on nitrocellulose membranes. After blocking and washing, the membranes were incubated with different concentrations of biotinylated-aptamer in 2-fold dilutions (7.5–300 nM), then washed and treated with HRP-streptavidin. The chemiluminescence intensity was calculated using ImageJ software. The K_d_ was determined with the $Y=Bmax\frac{X}{Kd+X}$ equation based on a nonlinear regression as previously described [10, 16].

### 2.9 Data interpretation

Data analysis was conducted using the Prism-GraphPad software (Version 8.4.3, California, USA), and a P-value (P) < 0.05 was considered statistically significant.

## 3 Results

### 3.1 Selection and enrichment of aptamers

**Figure 2:**
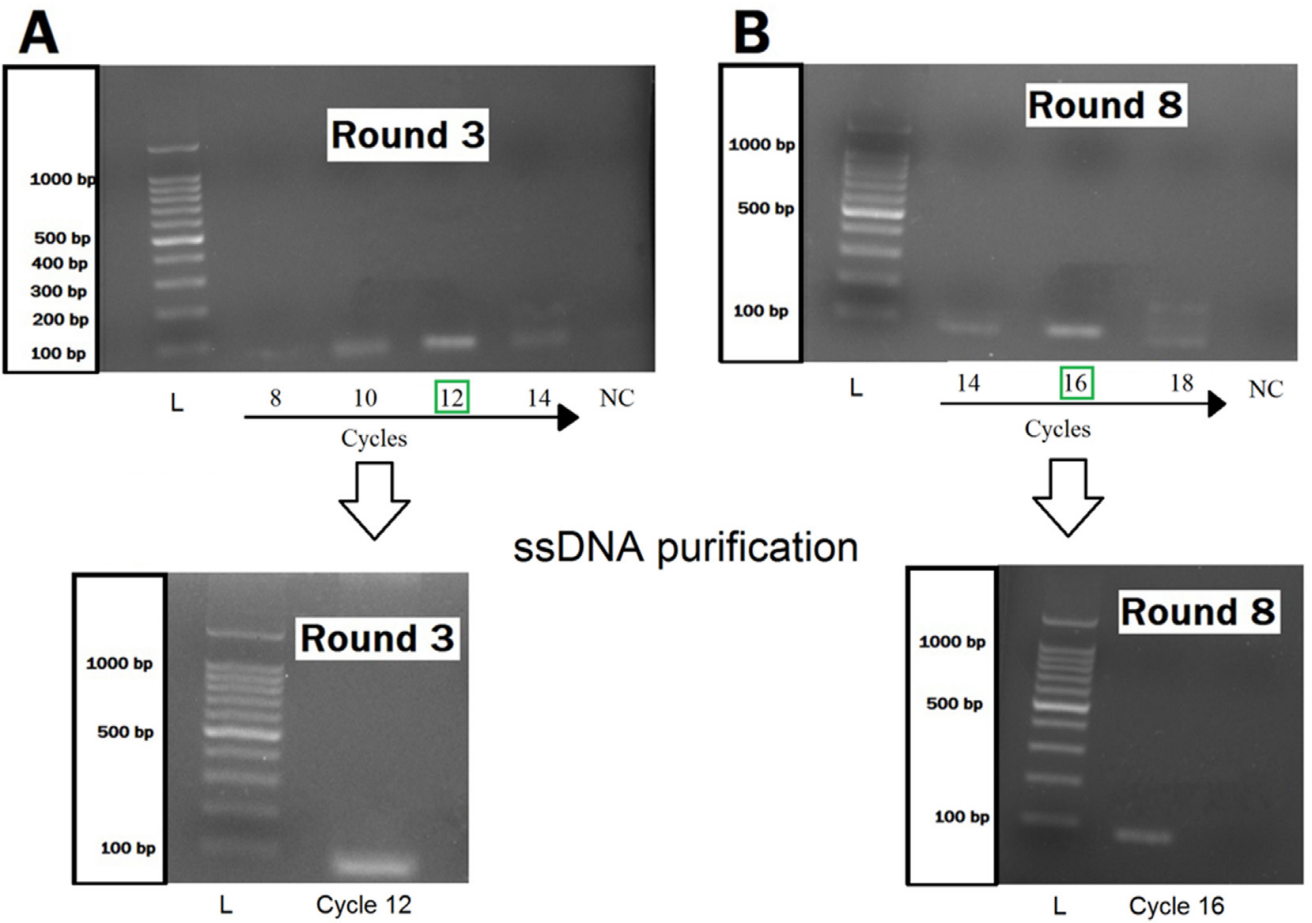
The enrichment pools with different PCR cycles: The enrichment pools of each round occurred in different PCR cycles. Desired cycles for 3^th^ and 8^th^ rounds were in the 12^th^ and 16^th^ cycles, respectively (A and B). After a determined desired round, the ssDNA purification was performed. NC: Negative Control. L: DNA Ladder.

Expressed mIL-2 protein with a His-tag domain was detected by SDS-PAGE as a monomeric form (23 kDa) at a concentration of 300 μg/ml (Supplementary 1). The gel electrophoresis results indicated that the enrichment pools of each round occurred in different PCR cycles (Figure 2, Table 1). To start the next round, dsDNAs converted into the ssDNAs. The gel electrophoresis results of the preparation and purification of ssDNA showed that the degradation of dsDNA with lambda exonuclease enzyme is suitable with high efficacy for the SELEX rounds (Supplementary 2).

### 3.2 SELEX round screening and aptamer sequencing

**Figure 3:**
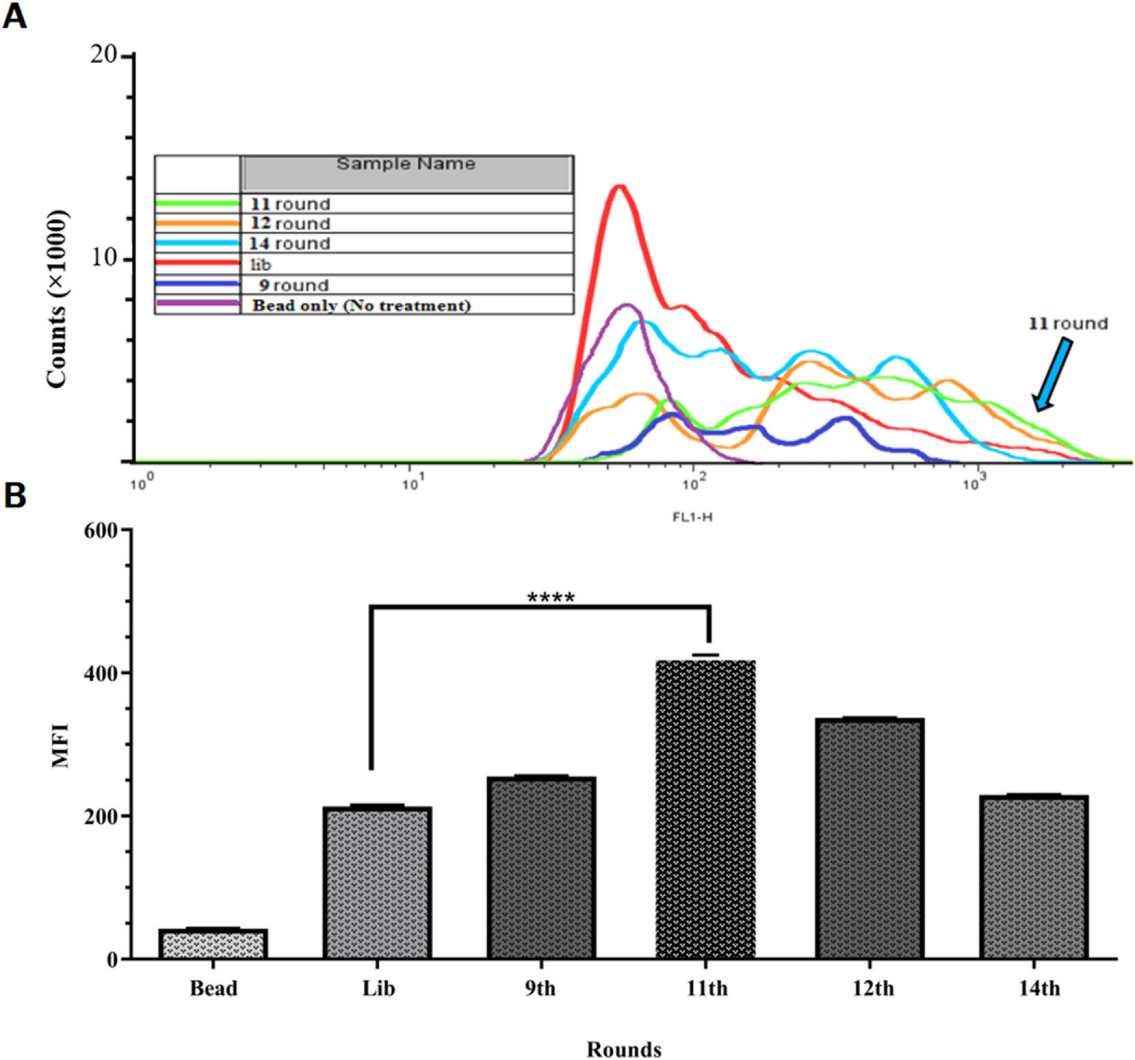
Round screening and MFI analysis: The flow cytometry MFI analysis of eluted pools showed that the MFI of the eleventh round was higher compared to rounds of library, 9^th^, 12^th^, and 14^th^ (A) with statistical differences (B).

In the current study, high-specific DNA aptamers were identified against mIL-2 after 14 SELEX rounds. The enrichment of aptamer pools was monitored by the flow cytometry method. The flow cytometry MFI showed that the MFI of the eleventh round was higher compared to rounds of 9^th^, 12^th^, 14^th^, and library (Figure 3A), with statistical differences (Figure 3B). The 11^th^ round of aptamers was cloned, and 35 clones were chosen. The gel electrophoresis results indicated that a few clones had junk insertions (Supplementary 3). The junked insertion was excluded, and 32 plasmids with proper insertion were included for nucleotide sequencing.

### 3.3 Structures of aptamers and specific affinity

**Figure 4:**
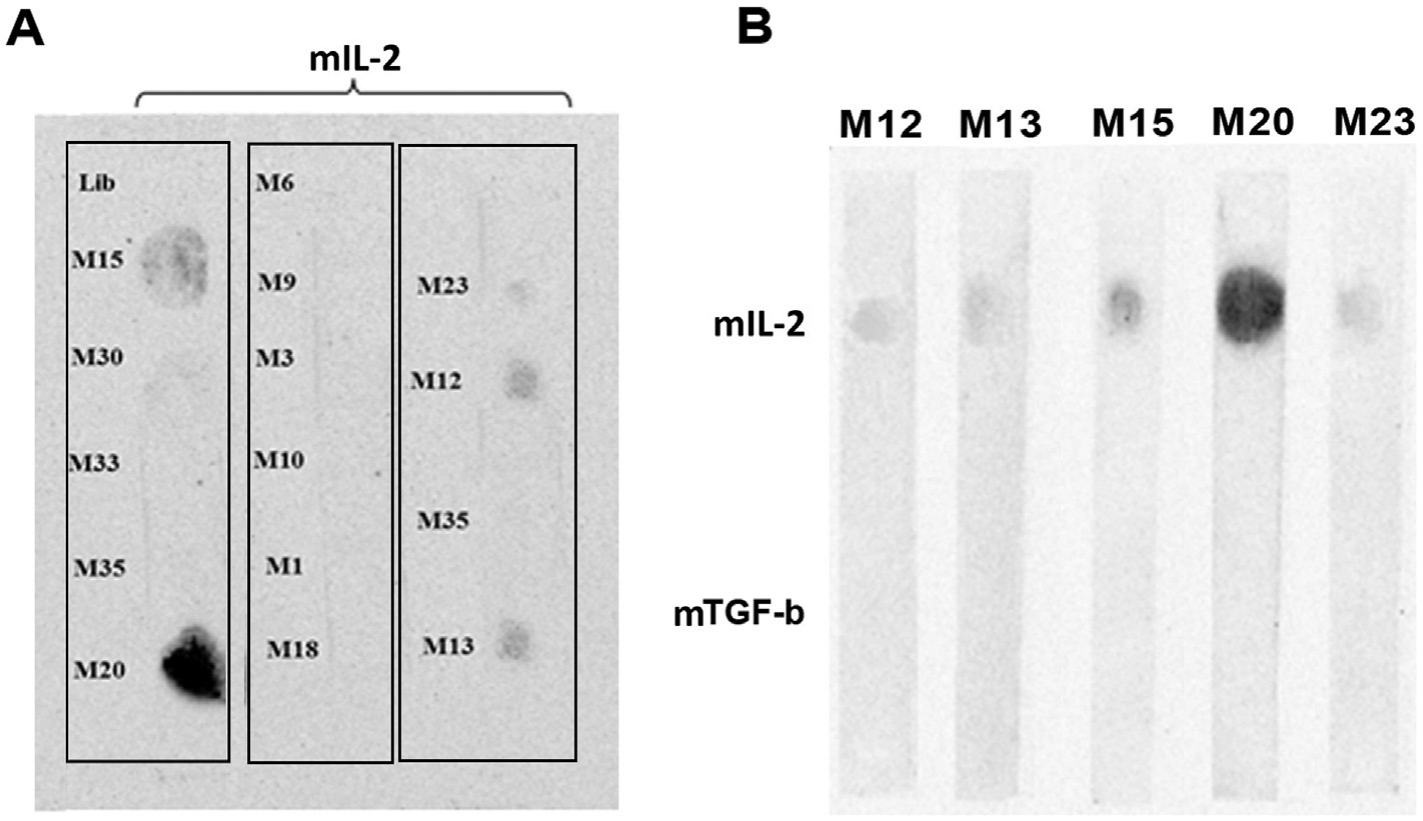
Dot-blot assay of the selected aptamer: 15 aptamers were evaluated for binding affinity and specificity (A). Five aptamers named M12, M13, M15, M20, and M23 had higher affinity to mIL-2. The M15 and M20 aptamers bound to the target with high affinity compared to other aptamers, and neither aptamer nonreacted with His-tagged recombinant mouse TGF-β (B).

**Figure 5:**
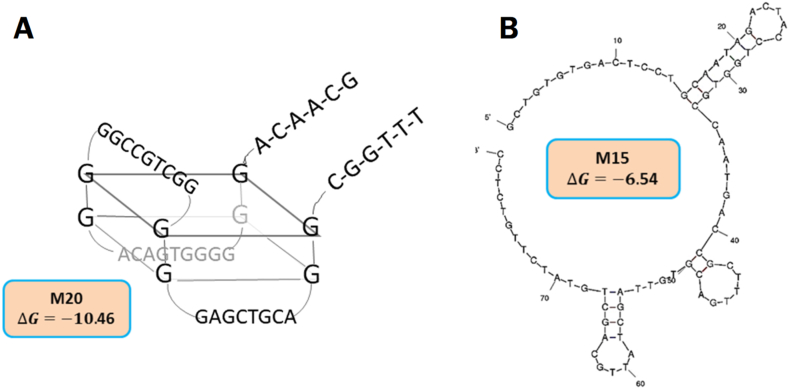
M15 and M20 structures: The M20 aptamer sequence had binary repetitions of guanine nucleotides and is probable to form into the G-quadruplex structure. So, its structure was predicted through QGRS Mapper software. According to DNA folding software, M20 and M15 aptamers had G-quadruplex (A) and stem-loop (B) structures, respectively.

The sequences of 32 aptamers were aligned by BioEdit software and divided based on similarities and differences (Supplementary 4). Then, the two-dimensional structure of aptamers was analyzed with the online M-fold software. The M-fold results predicted that 15 aptamers have stem, loop, and stem-loop structures with sufficient stability Supplementary 5), and other sequences did not have sufficient stability or structure. These 15 aptamers were evaluated for binding affinity and specificity based on the secondary structures. The results indicated that five aptamers named M12, M13, M15, M20, and M23 had a higher affinity for mIL-2 (Figure 4A). Finally, the dot blot results showed that the M15 and M20 aptamers bind to the target with high affinity compared to other aptamers. Neither aptamer reacted with His-tagged recombinant mouse TGF-β (Figure 4B). The M20 aptamer sequence had binary repetitions of guanine nucleotides and is probable to form into the G-quadruplex structure. So, the structure and G-scores of M20 were calculated and predicted through QGRS Mapper software (http://bioinformatics.ramapo.edu/QGRS). According to DNA folding software, M15 and M20 aptamers had stem-loop and G-quadruplex structures, respectively (Figure 5).

### 3.4 Aptamer K_d_

**Figure 6:**
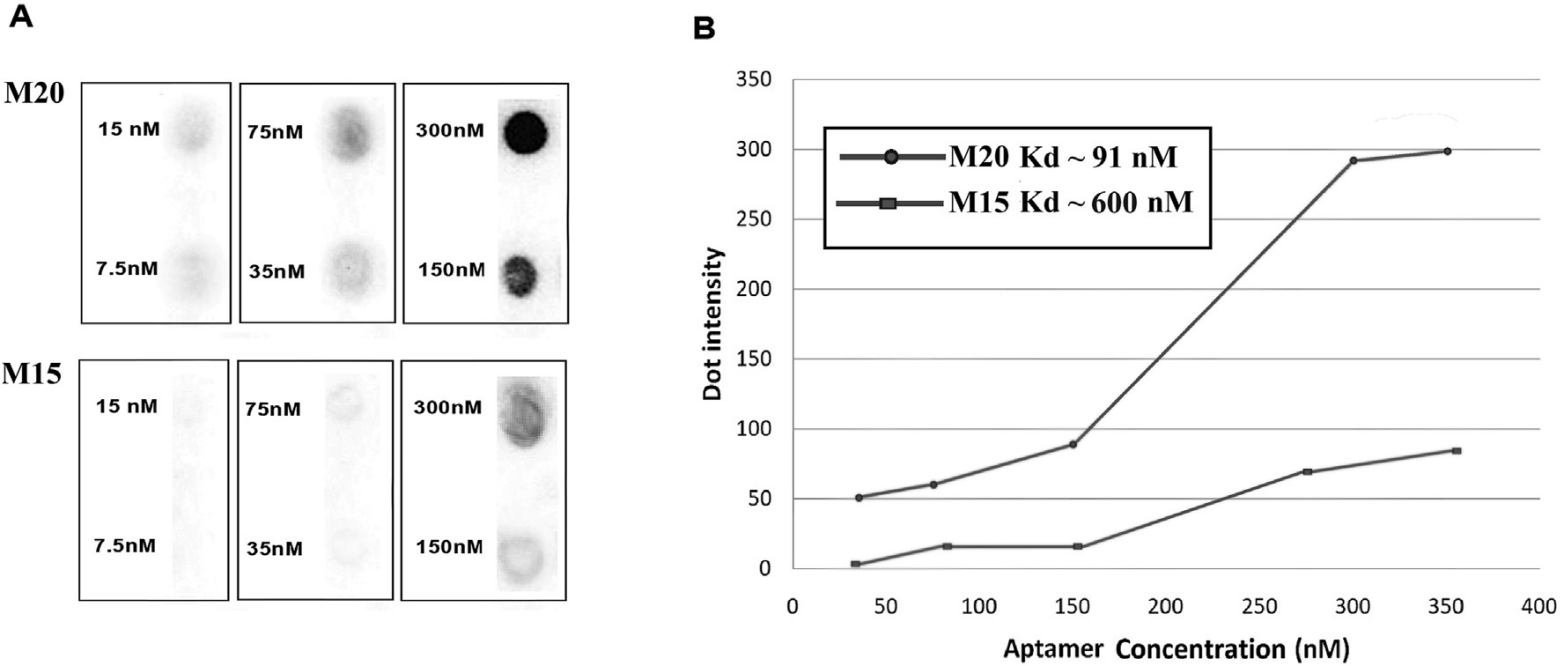
Anti-mIL-2 aptamer K_d_: With increasing serial dilution, the M20 aptamer showed a higher affinity to mIL-2 than M15 (A). The calculation of the dissociation constant predicted 91 and 600 nM K_d_ for M20 and M15, respectively (B).

In the current study, the K_d_ was calculated with the $\text{Y}=\text{Bmax}\frac{\text{X}}{\text{Kd}+\text{X}}$ equation from chemiluminescence intensities based on nonlinear regression analysis as previously described [10, 16]. Briefly, [Y] is the mean chemiluminescence intensity. The [Bmax] and [x] were considered concentrations of each aptamer that saturates 100 and 50 percent of binding sites, respectively. The results indicated that the affinity of the M20 aptamer was greater than the M15, and its predicted K_d_ was 91 nM (Figure 6).

## 4 Discussion

This study identified a G-quadruplex aptamer M20 that binds to the IL-2 cytokine with high affinity (K_d_: 91 nM) and specificity. It can be used to detect and recognize this cytokine in a variety of aptamer-based assays. Cytokines are among the most important immune system proteins that activate and inhibit different cells. Various studies have targeted these cytokines and their receptors with various molecules such as antibodies and aptamers [17, 18]. Moreover, the chemical synthesis of aptamers is very simple and cost-effective and can be used as an appropriate alternative to antibodies in antibody-based immune assays to detect cytokines. Moreover, the production of aptamers could be performed against a lower amount of target protein (in micrograms) in comparison with antibodies, which need an amount of large protein (in milligrams) [10]. Aptamer production is carried out in a variety of processes with SELEX techniques against proteins [19] and peptides [20] and with the Cell-SELEX technique against cell surface receptors or antigens [21, 22, 23, 24].

In the present study, after performing 14 rounds of SELEX, we selected high affinity and specific aptamers against mIL-2 protein from the 11^th^ round. To this aim, a protein-SELEX method was performed with increased-successive through rounds stringency, and after eleven rounds, Fifteen specific aptamers were selected. After more analysis, it was found that only two aptamers named M15 and M20 were bound to the mIL-2 with high affinity. The round stringency was applied to identify highly specific aptamers after a few rounds. In terms of round stringency, Prodeus *et al.* reached the two specific aptamers named M49 and M52 with high affinity against CD200R1 after 15 rounds. They considered round stringency on the slow reduction of target concentration [25]. In another study by Hui *et al.* for the identification of anti-DC-SIGN aptamers, 4 particular aptamers were identified after eleven rounds, one of which with a high affinity (about K_d_: 22 nM) was able to inhibit DCs interactions with ECs [26]. Like our results, they considered round stringency on aptamer/target incubation time and the amount of target proteins. In addition to the mentioned items, we performed counter SELEX on His-tagged protein and naked magnetic beads to eliminate non-specific aptamers and used Tween-20 to eliminate weak aptamers in the aptamer washing buffer. In the current study, the aptamer/protein ratio was 4:1 at the first SELEX round, and the mILL-2 concentration decreased gradually in SELEX progress. These strategies result in selected high-specific and -affinity aptamers in early SELEX rounds [27, 28, 29, 30].

We noticed that the number of cycles was increased with round progress, so enrichment of the pool occurred with fewer PCR cycles in early rounds. It seems that this result demonstrated decreasing non-specific aptamers in the SELEX process. In this regard, Hui *et al.* found that increasing PCR cycles from 8 to 24 occurred while identifying anti-DC-SIGN aptamers [26]. Also, in our previous study for identifying anti-human TNF-α aptamer, the increasing PCR cycles were observed from 12 to 22 cycles [10]. Moreover, the PCR cycles were increased from 12 to 18 cycles in our study. Also, we used the lambda exonuclease method to prepare single-strand aptamers in each round. This protocol, in comparison with other methods such as biotinylation [31] or asymmetric PCR [32], has high efficacy and is easy to use [33]. Furthermore, the number of SELEX rounds should be monitored in the SELEX process because with increasing SELEX process, due to the Taq-polymerase errors, which result in the identification of unspecific aptamers [34, 35, 36]. This event was observed in the 12^th^ and 14^th^ rounds, which caused decreasing MFI along with round progress (Figure 3). Also, we observed this event in two of our previous studies that were performed to identify anti-DC and anti-human TNF-α aptamers [10, 24, 37]. For this purpose, several methods are employed to monitor SELEX rounds, such as ELONA [38], dot blot [39], real-time PCR [10], and flow cytometry [24].

G-quadruplex aptamers constitute a unique class of G-rich and short single-stranded oligonucleotides capable of binding to various targets with high specificity and affinity. These aptamers are stabilized through stacking interactions of four guanines, which are assembled into a planar arrangement by Hoogsteen hydrogen bonding. These small molecules are highly polymorphic structures with various folding topologies, which could be considered a suitable alternative for antibodies to target biomolecules. These unique properties of G-quadruplexes’ aptamers depend on their structural and chemical nature, ease of chemical modification, increased cellular uptake, resistance to numerous serum nucleases, high stability, and convenient storage [40, 41]. The G-quadruplex aptamer was first reported in NMR studies on anti-thrombin aptamer [42]. The presence of cations such as Mg^2+^, Ca^2+^, Na^+,^ and K^+^ in the SELEX process, could increase the chance of G-quadruplex aptamer identification [43]. We used various cations in aptamer binding and washing buffers to preserve G-quadruplex aptamers through the SELEX process. The M20 aptamer sequence has binary repetitions of guanine nucleotides and is possibly formed into the G-quadruplex structure. The QGRS Mapper software predicted M20'd folding as a G-quadruplex structure. Also, the dot blot analysis revealed that the M20 is more stable compared to the M15 with a stem-loop structure.

The selection and production of this aptamer with high specificity and sensitivity for IL-2 is an important step in the development of aptamer immune-assay methods, which are more cost-effective, simple, faster, and more reliable than antibody-based immune-assay methods. According to a study by Low *et al., the* aptamer production cost is 10–50 times lower than the antibody. Therefore, the future perspective is more toward the use of aptamers for diagnosis or therapy in different diseases [9].

## 5 Conclusion

In this study, a simple SELEX protocol with high stringency was explained for the identification of DNA aptamers against protein targets, which resulted in the selection of high-specific and -affinity aptamers. The most critical factors in round stringency are aptamer/target incubation time, washing steps, and the amount of target proteins. Accordingly, we identified an anti-IL-2 G-quadruplex aptamer that binds to IL-2 with high affinity. An anti-IL-2 aptamer reported here has potential diagnostic and/or therapeutic application for IL-2-related disorders.

## Declarations

### Author contribution statement

Mohsen Momeni: Performed the experiments; Analyzed and interpreted the data; Contributed reagents, materials, analysis tools or data; Wrote the paper.

Kazem Mashayekhi, Jamshid Gholizadeh Navashenaq: Analyzed and interpreted the data; Wrote the paper.

Mojtaba Sankian: Conceived and designed the experiments; Contributed reagents, materials, analysis tools or data; Wrote the paper.

### Funding statement

Dr. Mojtaba Sankian was supported by 10.13039/501100004748Mashhad University of Medical Sciences [941436].

### Data availability statement

Data will be made available on request.

### Declaration of interests statement

The authors declare no conflict of interest.

### Additional information

No additional information is available for this paper.
