# Supplementary material for: Identification of G-quadruplex anti-Interleukin-2 aptamer with high specificity through SELEX stringency
Source: Heliyon. 2022 Jun 15;8(6):e09721. doi: 10.1016/j.heliyon.2022.e09721 (PMC9218155; doi:10.1016/j.heliyon.2022.e09721)
Supplement: Supplementary file.pdf [file mmc1.pdf]

Supplementary data

Supplementary data for research article entitled: '**Identification of G-quadruplex anti-Interleukin-2 aptamer with high specificity through SELEX stringency**' by Mohsen Momeni, Kazem Mashayekhi, Jamshid Gholizadeh Navashenaq, Mojtaba Sankian.

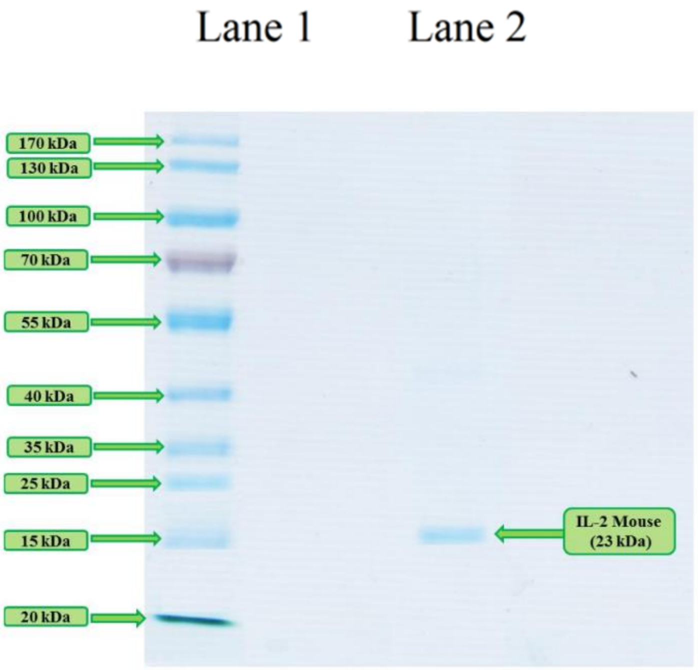

Supplementary 1: The SDS-PAGE analysis of mIL-2: The mIL-2 protein was expressed as a 23 kDa monomeric form with His-tag domain at a concentration of 300 µg/ml. Lane 1: low molecular Ladder, Lane 2: mIL-2.

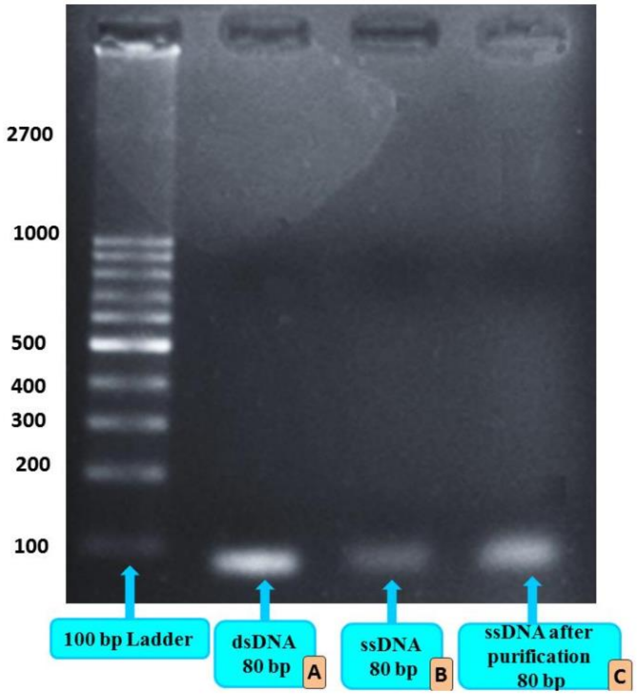

Supplementary 2: The ssDNA preparation and purification: The phosphorylated dsDNA pools (A) were digested by lambda exonuclease (B) and then purified (C). In this regard, the lambda exonuclease method has proper efficacy for the SELEX process. L: DNA Ladder.

A

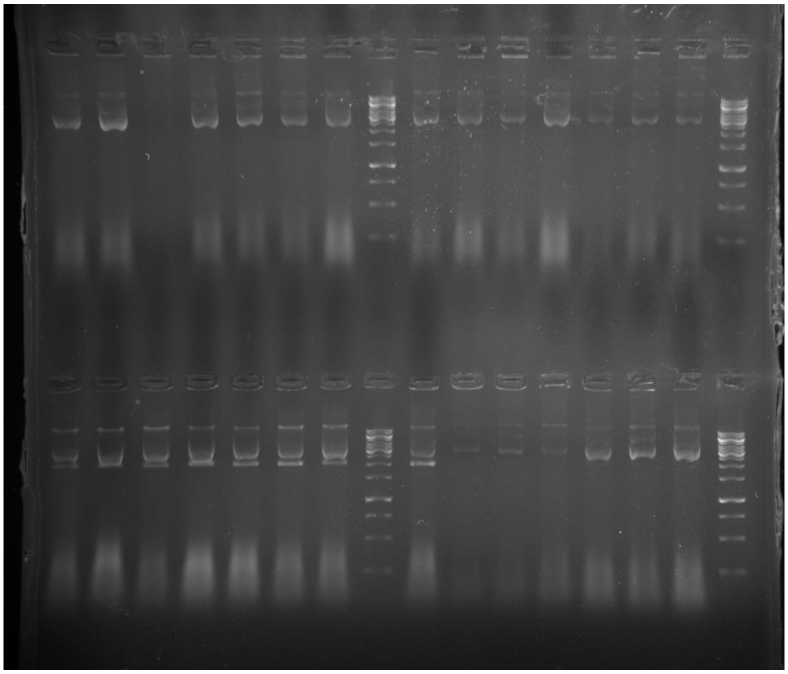

B

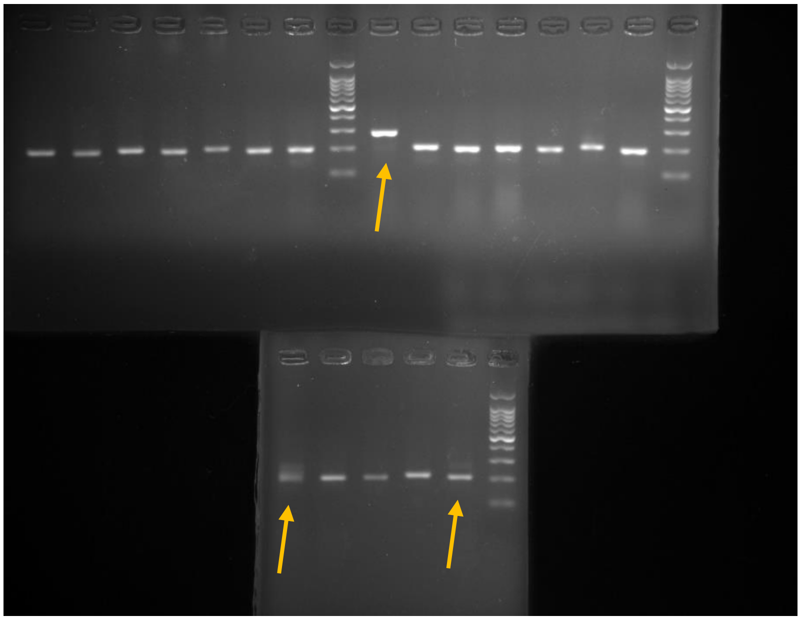

Supplementary 3: Recombinant plasmid extraction from 35 clones: The recombinant plasmids were extracted (A, 28 samples are shown). The PCR was performed with M13 and T7 primers to confirming insertion (B, 19 samples are shown), and some plasmids have had junk insertions (B, yellow arrows).

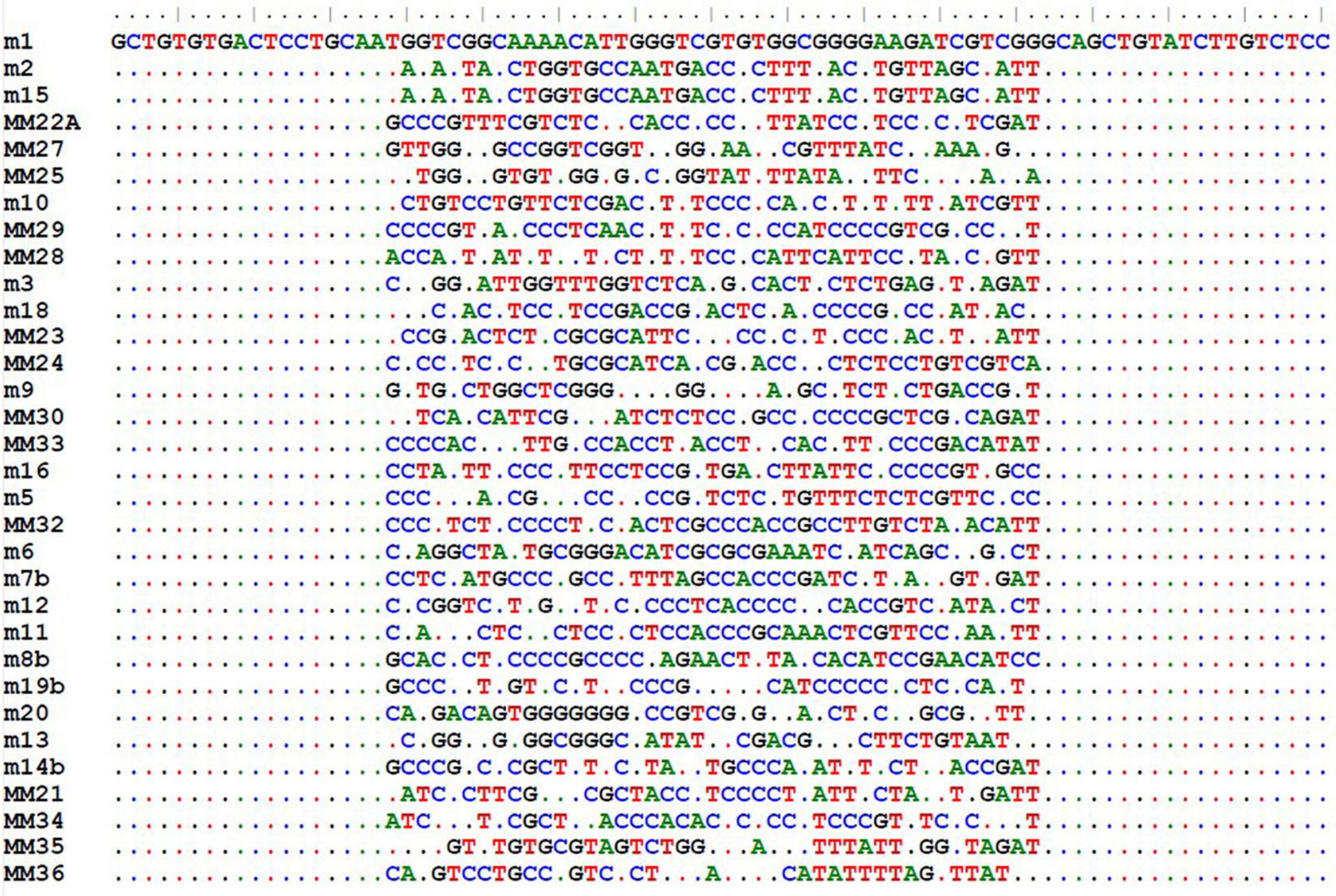

Supplementary 4: The list of the 32 sequences acquired after the cloning.

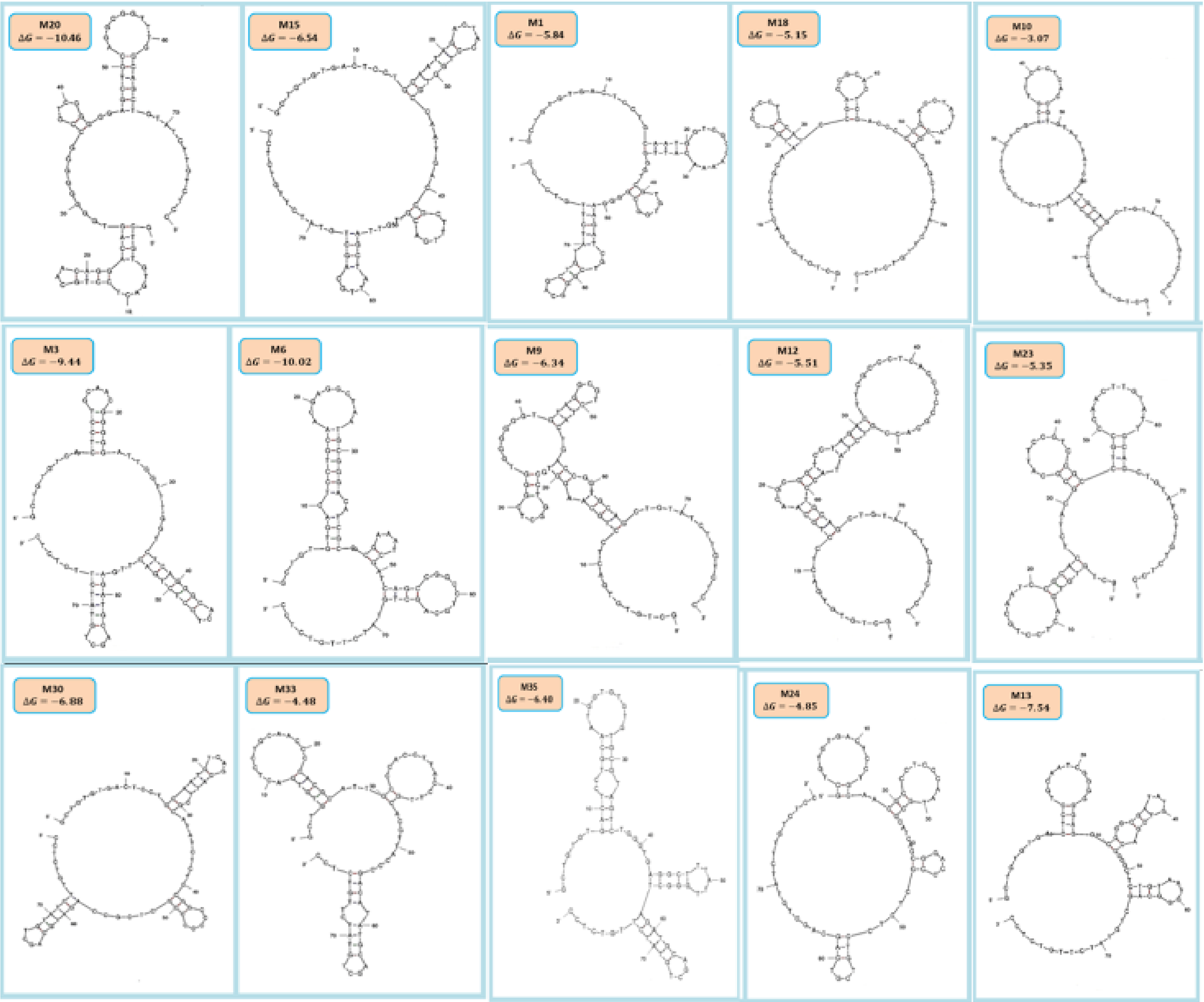

Supplementary 5: Prediction of aptamer structures: The sequences of 32 aptamers were aligned by BioEdit software and divided based on similarities and differences. Then, the aptamer 2D structures were determined with the M-fold software. The M-fold results predicted that 15 aptamers have a stem, loop, and stem-loop structures with sufficient stability.
